# Analytical solutions for systems of partial differential–algebraic equations

**DOI:** 10.1186/2193-1801-3-137

**Published:** 2014-03-10

**Authors:** Brahim Benhammouda, Hector Vazquez-Leal

**Affiliations:** Higher Colleges of Technology, Abu Dhabi Men’s College, P.O. Box 25035, Abu Dhabi, United Arab Emirates; Electronic Instrumentation and Atmospheric Sciences School, Universidad Veracruzana, Cto. Gonzalo Aguirre Beltrán S/N, 91000 Xalapa, Mexico

**Keywords:** Partial differential-algebraic equations, Power series method, Laplace transform, Padé approximant; Analytical solutions

## Abstract

This work presents the application of the power series method (PSM) to find solutions of partial differential-algebraic equations (PDAEs). Two systems of index-one and index-three are solved to show that PSM can provide analytical solutions of PDAEs in convergent series form. What is more, we present the post-treatment of the power series solutions with the Laplace-Padé (LP) resummation method as a useful strategy to find exact solutions. The main advantage of the proposed methodology is that the procedure is based on a few straightforward steps and it does not generate secular terms or depends of a perturbation parameter.

## Introduction

As widely known, the importance of research on partial differential-algebraic equations (PDAEs) is that many phenomena, practical or theoretical, can be easily modelled by such equations. Those kinds of equations arise in fields like: nanoelectronics (Bartel and Pulch 2006), electrical networks (Ali et al. 2005,2003; Günther 2000) and mechanical systems (Simeon 1996), among others.

In recent years, PDAEs have received much attention, nevertheless the theory in this field is still young. For linear PDAEs the convergence of Runge-Kutta method is investigated in (Strehmel and Debrabant 2005). The numerical solution of linear PDAEs with constant coefficients and the study of indices are given in (Lucht et al. [Bibr CR31][Bibr CR32]; Lucht and Strehmel 1998; Lucht et al. 1999). Linear and nonlinear PDAEs are characterized by means of indices which play an important role in the treatment of these equations. The differentiation index is defined as the minimum number of times that all or part of the PDAE must be differentiated with respect to time, in order to obtain the time derivative of the solution, as a continuous function of the solution and its space derivatives (Martinson and Barton 2000).

Higher-index PDAEs (differentiation index greater than one) are known to be difficult to treat even numerically. Often such problems are first transformed to index-one systems before applying numerical integration methods. This procedure called index-reduction, can be very expensive and may change the properties of the solution. Since applications problems in science and engineering often lead to higher-index PDAEs, new techniques are required to solve these problems efficiently.

Modern methods like homotopy perturbation method (HPM) (He 1999,2000,1998; Vazquez-Leal et al. 2012), homotopy analysis method (HAM) (Guerrero et al. 2013), variational iteration method (VIM) (Khan Y, et al. 2012), generalized homotopy method (Vazquez-Leal 2013), among others, are powerful tools to approximate nonlinear and linear problems. The HPM has been successfully applied to solve various kinds of nonlinear problems in science and engineering, including Volterra’s integro-differential equation (El-Shahed 2005), nonlinear differential equations (He 1998), nonlinear oscillators (He 2004), partial differential equations (PDEs) (He 2005a), bifurcation of nonlinear problems (He 2005b) and boundary-value problems (He 2006). Recently, the modifications of the HPM have been used to solve DAEs (Aminikhah and Hemmatnezhad 2011; Asadi et al. 2012; Salehi et al. 2012; Soltanian et al. 2010). Nevertheless, the power series method (PSM) (Forsyth 1906; Ince 1956) is a well-known classic straightforward procedure from literature that can be applied successfully to solve differential equations of different kind: linear ordinary differential equations (ODEs) (Coddington 1989; Forsyth 1906; Ince 1956; Kreyszig 1999), nonlinear ODEs (Biazar et al. 2005; Fairen et al. 1988; Filipich and Rosales 2002; Filipich et al. 2004; Guzel and Bayram 2005; Kreyszig 1999) and linear PDEs (Kurulay and Bayram 2009), among others. This method establishes that the solution of a differential equation can be expressed as a power series of the independent variable.

In this paper we present the application of a hybrid technique combining PSM, Laplace Transform (LT) and Padé Approximant (PA) (Barker 1975) to find analytical solutions for PDAEs (Ebaid 2011; Gőkdoğan et al. 2012; Merdan et al. 2011; Momani and Ertűrk 2008; Momani et al. 2009; Sweilam and Khader 2009; Tsai and Chen 2010; Yamamoto et al. 2002). Solutions to PDAEs are first obtained in convergent series form using the PSM. To improve the solution obtained from PSM’s truncated series, we apply LT to it, then convert the transformed series into a meromorphic function by forming its PA. Finally, we take the inverse LT of the PA to obtain the analytical solution. This hybrid method (LPPSM), which combines PSM with Laplace-Padé post-treatment greatly improves PSM’s truncated series solutions in convergence rate. In fact, the Laplace-Padé resummation method enlarges the domain of convergence of the truncated power series and often leads to the exact solution.

It is important to remark that LPPSM can obtain exact solutions without requiring the index-reduction of the PDAEs. The proposed method does not produce noise terms also known as secular terms as the homotopy perturbation based techniques (Soltanian et al. 2010). This greatly reduces the volume of computation and improves the efficiency of the method in comparison to the perturbation based methods. What is more, LPPSM does not require a perturbation parameter as the perturbation based techniques including HPM. Finally, LPPSM is straightforward and can be programmed using computer algebra packages like Maple or Mathematica.

The rest of this paper is organized as follows. In the next section we illustrate the basic concept of the PSM. The main idea behind the Padé approximant is given in section “Padé approximant”. In section “Laplace-Padé resummation method”, we give the basic concept of the Laplace-Padé resummation method. The application of PSM to solve PDAE systems is depicted in section “Application of PSM to solve PDAE systems”. In section “Test problems”, we apply LPPSM to solve two PDAEs problems of index-one and index-three. In section “Discussion”, we give a brief discussion. Finally, a conclusion is drawn in the last section.

## Basic concept of power series method

It can be considered that a nonlinear differential equation can be expressed as1

having as boundary condition2

where *A* is a general differential operator, *f*(*t*) is a known analytic function, *B* is a boundary operator, and *Γ* is the boundary of domain *Ω*.

PSM (Forsyth 1906; Ince 1956) establishes that the solution of a differential equation can be written as3

where *u*_0_,*u*_1_,… are unknowns to be determined by series method.

The basic process of series method can be described as:

1. Equation (3) is substituted into (1), then we regroup the equation in terms of powers of *t*.
2. We equate each coefficient of the resulting polynomial to zero.
3. The boundary conditions of (1) are substituted into (3) to generate an algebraic equation for each boundary condition.
4. Aforementioned steps generate an algebraic linear system for the unknowns of (3).
5. Finally, we solve the algebraic linear system to obtain the coefficients *u*_0_,*u*_1_,…

## Padé approximant

Given an analytical function *u*(*t*) with Maclaurin’s expansion4

The Padé approximant to *u*(*t*) of order [ *L*,*M*] which we denote by [ *L*/*M*]_*u*_(*t*) is defined by (Barker 1975)5

where we considered *q*_0_=1, and the numerator and denominator have no common factors.

The numerator and the denominator in (5) are constructed so that *u*(*t*) and [ *L*/*M*]_*u*_(*t*) and their derivatives agree at *t*=0 up to *L*+*M*. That is6

From (6), we have7

From (7), we get the following algebraic linear systems8

and9

From (8), we calculate first all the coefficients *q*_*n*_, 1≤*n*≤*M*. Then, we determine the coefficients *p*_*n*_, 0≤*n*≤*L* from (9).

Note that for a fixed value of *L*+*M*+1, the error (6) is smallest when the numerator and denominator of (5) have the same degree or when the numerator has degree one higher than the denominator.

## Laplace-Padé resummation method

Several approximate methods provide power series solutions (polynomial). Nevertheless, sometimes, this type of solutions lacks of large domains of convergence. Therefore, Laplace-Padé (Ebaid 2011; Gőkdoğan et al. 2012; Merdan et al. 2011; Momani and Ertűrk 2008; Momani et al. 2009; Sweilam and Khader 2009; Tsai and Chen 2010; Yamamoto et al. 2002) resummation method is used in literature to enlarge the domain of convergence of solutions or inclusive to find exact solutions.

The Laplace-Padé method can be explained as follows:

1. First, Laplace transformation is applied to power series (3).
2. Next, *s* is substituted by 1/*t* in the resulting equation.
3. After that, we convert the transformed series into a meromorphic function by forming its Padé approximant of order [ *L*/*M*]. *L* and *M* are arbitrarily chosen, but they should be of smaller value than the order of the power series. In this step, the Padé approximant extends the domain of the truncated series solution to obtain better accuracy and convergence.
4. Then, *t* is substituted by 1/*s*.
5. Finally, by using the inverse Laplace *s* transformation, we obtain the exact or approximate solution.

## Application of PSM to solve PDAE systems

Since many application problems in science and engineering are often modelled by semi-explicit PDAEs, we consider therefore the following class of PDAEs1011

where *u*_*k*_: , *k*=1,2 and *b*>*a*.

System (10)-(11) is subject to the initial condition12

and some suitable boundary conditions13

where *g*(*x*) is a given function.

We assume that the solution to initial boundary-value problem (10)-(13) exists, is unique and sufficiently smooth.

To simplify the exposition of the PSM, we integrate first equation (10) with respect to *t* and use the initial condition (12) to obtain14

It is important to note that the time integration of equation (10) is not relevant to the solution procedure presented here, so one can apply the PSM directly to (10).

In view of PSM, we assume the solution components *u*_*k*_(*t*,*x*),*k*=1,2 to have the form15

where *α*_*k*,*n*_(*x*), *k*=1,2; *n*=0,1,2,… are unknown functions to be determined later on by the PSM.

Then substitute (15) into system (11)-(14) and equate the coefficients of powers of *t* in the resulting polynomial equations to zero to get an algebraic linear system for these coefficients. Finally, we use equation (15) to obtain the exact solution components *u*_*k*_, *k*=1,2 as series. The solutions series obtained from PSM may have limited regions of convergence, even if we take a large number of terms. Therefore, we apply the Laplace-Padé resummation method to PSM truncated series to enlarge the convergence region as depicted in the next section.

## Test problems

In this section, we will demonstrate the effectiveness and accuracy of the LPPSM presented in the previous section through two PDAE systems of index-one and index-three.

### Nonlinear index-one system

Consider the following nonlinear index-one PDAE which arises as a similarity reduction of Navier-Stokes equations (Budd et al. 1994)1617

where 0<*x*<1 and *t*>0.

System (16)-(17) is subject to the following initial condition18

and boundary conditions19

The exact solution of problem (16)-(19) is20

Since one time differentiation of equation (17) determines *u*_2*t*_ in terms of *u* and its space derivatives, then PDAE (16)-(17) is index-one. Note that no initial condition is prescribed for the variable *u*_2_ as this is determined by the PDAE.

In order to simplify the exposition of the PSM presented in section “Application of PSM to solve PDAE systems” to solve (16)-(17), we first integrate equation (16) with respect to *t* and use the initial condition (18) to get21

In view of the PSM, we assume the solution components *u*_*k*_,*k*=1,2 to have the form22

where *α*_*k*,*n*_(*x*),*k*=1,2; *n*=0,1,2,… are unknown functions to be determined later on by the PSM.

Then, we substitute (22) into equations (17) and (21) to get2324

where (^′^) denotes the ordinary derivative with respect to *x*.

Equating the coefficients of powers of *t* to zero in (24) then solving the resulting equation for *α*_2,*n*_(*x*) and using the boundary conditions (19), we have25

Now equation (23) can be written as a series26

where

Equating all coefficients of powers of *t* to zero in (26), yields *α*_1,0_(*x*)= cos*πx* and the recursive formula for *α*_1,*n*_(*x*)27

From recursion (27), we get *α*_1,1_(*x*)=-*π*^2^ cos*πx* and *α*_1,2_(*x*)=(*π*^4^/2) cos*πx*.

From equation (25), we get *α*_2,0_(*x*)=(1/*π*) sin*πx*, *α*_2,1_(*x*)=-*π* sin*πx* and *α*_2,2_(*x*)=(*π*^3^/2) sin*πx*. Using (22) and the coefficients recently obtained, we have28

and29

Similarly, the coefficients *α*_1,*n*_(*x*) and *α*_2,*n*_(*x*) for *n*≥3 can be found from (27) and (25) respectively.

The solutions series obtained from the PSM may have limited regions of convergence, even if we take a large number of terms. Accuracy can be increased by applying the Laplace-Padé post-treatment. First, we apply *t*-Laplace transform to (28) and (29). Then, we substitute *s* by 1/*t* and apply *t*-Padé approximant to the transformed series. Finally, we substitute *t* by 1/*s* and apply the inverse Laplace *s*-transform to the resulting expressions to get the approximate or exact solutions.

Applying Laplace transforms to *u*_1_(*t*,*x*) and *u*_2_(*t*,*x*) yields30

and31

For the sake of simplicity let *s*=1/*t*, then32

and33

All of the [ *L*/*M*]*t*-Padé approximants of (32) and (33) with *L* ≥1 and *M* ≥1 and *L*+*M*≤3 yield 34

and35

Now since *t*=1/*s*, we obtain  and  in terms of *s* as follows3637

Finally, applying the inverse LT to the Padé approximants (36) and (37), we obtain the approximate solution which is in this case the exact solution (20) in closed form.

### Linear index-three system

Consider the following index-three PDAE system383940

where 0<*x*<1 and *t*>0.

System (38)-(40) is subject to the following initial conditions4142

and the boundary conditions43

The exact solution of problem (38)-(43) is44

Since three time differentiations of equation (40) determine *u*_3*t*_ in terms of the solution *u* and its space derivatives, then PDAE (38)-(40) is index-three. Therefore, this PDAE is difficult to solve numerically. Moreover no initial condition is prescribed for the variable *u*_3_ as this is determined by the PDAE.

In order to simplify the exposition of the LPPSM presented in section “Application of PSM to solve PDAE systems” to solve (38)-(43), we first integrate equations (38) and (39) twice with respect to *t* and use the initial conditions (41)-(42) to get4546

In view of the PSM, we assume the solution components *u*_*k*_(*t*,*x*),*k*=1,2,3 to have the form47

where *α*_*k*,*n*_(*x*),*k*=1,2,3; *n*=0,1,2,… are unknown functions to be determined later on by the PSM.

Substituting (47) into equations (40), (45) and (46) we get the system4849

and50

where (^′^) denotes the ordinary derivative with respect to *x*.

System (48)-(50) can be rewritten as series51

Equating the coefficient of powers of *t* to zero in (51) then solving the resulting system we find the coefficients *α*_*k*,*n*_(*x*), for *k*=1,2,3 and *n*=0,1,2,…

and the nonsingular algebraic linear system for the unknown functions *α*_1,*n*_, *α*_2,*n*_ and *α*_3,*n*-2_52

Solving system (52) exactly, we obtain the recursions53

where .

For *n*=2,3,4, we have *δ*_*n*_(*x*)=0 and hence

and

Using (47) and the coefficients recently obtained, we get5455

and56

Similarly, the coefficients *α*_1,*n*_(*x*), *α*_2,*n*_(*x*) and *α*_3,*n*-2_(*x*) for *n*≥5 can be found from (53). The solutions series obtained from the PSM may have limited regions of convergence, even if we take a large number of terms. Therefore, we apply the *t*-Padé approximation technique to these series to increase the convergence region. First *t* -Laplace transform is applied to (54), (55) and (56). Then, *s* is substituted by 1/*t* and the *t*-Padé approximant is applied to the transformed series. Finally, *t* is substituted by 1/*s* and the inverse Laplace *s* -transform is applied to the resulting expressions to get the approximate or exact solutions.

Applying Laplace transforms to *u*_1_(*t*,*x*), *u*_2_(*t*,*x*) and *u*_3_(*t*,*x*) yields5758

and59

For the sake of simplicity let *s*=1/*t*, then6061

and62

All of the [ *L*/*M*] *t*-Padé approximants of (60), (61) and (62) with *L*≥1 and *M*≥1 and *L*+*M*≤3 yield6364

and65

Now since *t*=1/*s*, we obtain ,  and  in terms of *s* as follows6667

and68

Finally, applying the inverse Laplace transform to the Padé approximants (66), (67) and (68), we obtain the approximate solution which is in this case the exact solution (44) in closed form.

## Discussion

In this paper we presented the power series method (PSM) as a useful analytical tool to solve partial differential-algebraic equations (PDAEs). Two PDAE problems of index-one and index-three were solved by this method leading to the exact solutions. The method has successfully handled the index-three PDAE without the need for a preprocessing step of index-reduction. For each of the two problems solved here, the PSM transformed the PDAE into an easily solvable linear algebraic system for the coefficient functions of the power series solution. To improve the PSM solution, a Laplace-Padé (LP) post-treatement is applied to the PSM’s truncated series leading to the exact solution. Additionally, the solution procedure does not involve unnecessary computation like that related to noise terms (Soltanian et al. 2010). This greatly reduces the volume of computation and improves the efficiency of the method. It should be noticed that the high complexity of these problems was effectively handled by LPPSM method due to the malleability of PSM and resummation capability of Laplace-Padé. What is more, there is not any standard analytical or numerical methods to solve higher-index PDAEs, converting the LPPSM method into an attractive tool to solve such problems.

On one hand, semi-analytic methods like HPM, HAM, VIM among others, require an initial approximation for the sought solutions and the computation of one or several adjustment parameters. If the initial approximation is properly chosen the results can be highly accurate, nonetheless, no general methods are available to choose such initial approximation. This issue motivates the use of adjustment parameters obtained by minimizing the least-squares error with respect to the numerical solution.

On the other hand, PSM or LPPSM methods do not require any trial equation as requisite for the starting the method. What is more, PSM obtains its coefficients using an easy computable straightforward procedure that can be implemented into programs like Maple or Mathematica. Finally, if the solution of the PDAE is not expressible in terms of known functions then the LP post-treatement will provide a larger domain of convergence.

## Conclusion

This work presented LPPSM method as a combination of the classic PSM and a resummation method based on the Laplace transforms and Padé approximant. Firstly, the solutions of PDAEs are obtained in convergent series forms using PSM. Next, in order to enlarge the domain of convergence of the truncated power series, a post-treatment combining Laplace transform and Padé approximant is applied. This technique that we call LPPSM greatly improves PSM’s truncated series solutions in convergence rate, and often leads to the exact solution. Additionally, PSM is an attractive tool, because it does not require of a perturbation parameter to work and it does not generate secular terms (noise terms) as other semi-analytical methods like HPM, HAM or VIM.

By solving two problems, we presented the LPPSM as a handy tool with high potential to solve linear/nonlinear higher-index PDAEs. Additionally, the LPPSM does not require an index-reduction to solve higher-index PDAEs. Furthermore, we obtained successfully the exact solutions of such two problems highlighting the efficiency of LPPSM. What is more, the proposed method is based on a straightforward procedure, suitable for engineers. Finally, further research should be performed to solve other higher-index nonlinear PDAE systems.
